# Impact of cigarette smoke on osteogenic and osteoclast signaling in middle palatal suture

**DOI:** 10.1590/0103-6440202203966

**Published:** 2022-04-29

**Authors:** Maya Fernanda Manfrin Arnez, Patrícia Maria Monteiro, Francisco Wanderley Garcia Paula-Silva, Gabriel Barretto Dessotti, Luciane Macedo de Menezes, Erika Calvano Küchler, Sandra Yasuyo Fukada Alves, Mirian Aiko Nakane Matsumoto, Maria Bernadete Sasso Stuani

**Affiliations:** 1 Department of Othodontic, School of Dentistry of Ribeirao Preto, University of Sao Paulo, Ribeirao Preto, Sao Paulo, Brazil.; 2 Department of Clinical Analysis, Toxicology and Food Science, School of Pharmaceutical Sciences of Ribeirão Preto, University of Sao Paulo, Ribeirao Preto, Sao Paulo, Brazil.; 3 Department of Orthodontics, School of Dentistry, Pontifical Catholic University of Rio Grande do Sul, Porto Alegre, Brazil.; 4 Department of Orthodontics, School of Dentistry, University Medical Centre of Regensburg, Germany.; 5 Department of Physics and Chemistry, School of Pharmaceutical Sciences of Ribeirão Preto, University of Sao Paulo, Ribeirao Preto, Sao Paulo, Brazil.

**Keywords:** cigarette smoke, middle palatal suture, children

## Abstract

Considering that smoking is a public health problem that has been growing among adolescents, the aim of this study was to investigate the impact of cigarette smoke on osteogenic and osteoclastogenic signaling in middle palatal suture of rats. Male Wistar rats exposed (n = 30) or not to cigarette smoke (n = 30) were used. Exposure to smoke was carried out for two daily periods of 3 minutes each, with an interval of 12 hours between exposures. After the experimental periods of 3, 7, 14 and 21 days, the animals were euthanized. The collected tissues were analyzed using light microscopy and real-time RT-PCR was performed to investigate gene expression. The data obtained were compared using the Kruskal Wallis and Dunn tests (⍺ = 5%). Morphologically, there were no significant changes in the middle palatal suture of rats exposed or not to cigarette smoke during 3, 7, 14 and 21 days (p> 0.05). On the other hand, osteoclastogenic signaling was increased in animals exposed to smoke and was characterized by a higher production of RANKL at 3 and 14 days (p <0.05), with no change in the synthesis of RANK and osteoprotegerin (p> 0.05). Interestingly, in the exposed animals, an early increase in the synthesis of osteocalcin, bone sialoprotein and osteopontin was also identified at 3 days of exposure (p <0.05), not sustained over time (p> 0.05). Cigarette smoke modulates osteogenic and osteoclastogenic signaling in the middle palatal suture of young rats, although morphological changes have not been evidenced.

## Resumo

Considerando que a fumaça de cigarro é um problema de saúde pública que está crescendo entre os adolescentes, o objetivo deste estudo foi investigar o impacto da fumaça de cigarro na sinalização osteogênica e osteoclastogênica da sutura palatina mediana de ratos. Foram utilizados ratos Wistar machos expostos (n=30) e não expostos à fumaça de cigarro (n=30). A exposição à fumaça de cigarro foi realizada duas vezes ao dia por 3 minutos, com um intervalo de 12 horas entre as exposições. Os animais foram mortos após o período experimental de 3, 7, 14 e 21 dias. Os tecidos coletados foram analisados em microscópico de luz e pelo RT-PCR em tempo real foi realizado para investigar a expressão gênica. s dados obtidos foram comparados usando o testes de Kruskal Wallis e Dunn (⍺ = 5%). Morfoligicamente, não houve mudança significativa na sutura palatina mediana nos ratos expostos ou não à fumaça de cigarro durante os tempo de 3, 7, 14 e 21 dias (p> 0.05). Por outro lado, a sinalização osteogênica esta aumentada nos animais expostos à fumaça e foi caracterizado por um aumento da produção de RANKL aos 3 e 14 dias (p <0.05), sem mudança na síntese da produção de RANK e osteoprotegerina (p> 0.05). Curiosamente, nos animais expostos, também foi observado um aumento precoce da síntese de osteocalcina, sialoproteína óssea e de osteopontina aos 3 dias de exposição, o que não foi mantido ao longo do tempo. A fumaça de cigarro modula a sinalização osteogênica e osteoclastogênica na sutura palatina mediana de ratos jovens, apesar de não tenha sido evidenciado alterações morfológicas.

## Introduction

Smoking is considered a pediatric disease by the World Health Organization (WHO). Recent data show that one third of young Brazilians try cigarettes before 12 years of age. It is estimated that between 82,000 and 99,000 young people worldwide start smoking each day. In Brazil, this public health problem has been growing dramatically among schoolchildren, whose highest rates are among adolescents. Of the experimenters, 50% will become regular smokers in adulthood ^1^. It was estimated that more than 600,000 non-smokers die each year in the world due to passive exposure to smoking and that 28% of these deaths are children ^2^.

The literature presents several exogenous factors and biological events that alter the local level of chemical mediators that affect bone remodeling in the oral cavity. The different components of the cigarette appear to have a major negative impact on the bone remodeling ^3^. Nicotine, carbon monoxide and cyanide are the elements of cigarette smoke that are commonly related to an impaired tissue repair process ^4^. Other cigarette components, such as acrolein and acetaldehyde have shown in vitro a detrimental effect on the proliferation and adhesion of important cells involved on the healing process, such as fibroblasts ^5^. Long-term cigarette smoking is associated with reduced bone mineral density in osteoporosis and increased fracture risk ^6^. Nonetheless, the impact of cigarette smoke exposure on the different mediators that regulate the process of osteogenesis and osteoclastogenesis in the middle palatal suture of young people is not known.

Cellular and molecular events that occur during osteogenesis and osteoclastogenesis have been extensively investigated in order to understand the process of bone formation (anabolism) and resorption (catabolism). Bone remodeling is orchestrated by cells of the osteoblastic (mesenchymal) and osteoclastic (hematopoietic) lineage involving a complex network of cell-cell, matrix-cell interactions and multiple cell signaling pathways, along with the participation of systemic hormones, growth factors , production of local cytokines, which act in an autocrine and paracrine manner ^7^ (Arnez et al., 2017). These molecules can stimulate many cellular responses through different types of cells in bone tissue, thus providing a favorable microenvironment for tissue deposition or reabsorption ^8^.

Osteoclastogenesis and bone resorption are controlled by the RANK (nuclear factor kappa B activator receptor), RANKL (RANK ligand) and OPG (osteoprotegerin) system ^9^. RANKL is an important molecule for the differentiation of hematopoietic progenitor cells into mature osteoclasts and exerts its effects through its binding to the RANK receptor. On the other hand, OPG is a soluble receptor secreted as a RANKL antagonist, since it blocks this activation by directly binding to RANKL avoiding the bone resorption ^9^ ^,^ ^10^ ^,^ ^11^. On the other hand, other mediators are related to osteogenesis, i.e., bone formation, such as osteocalcin, bone sialoprotein, osteopontin, osteonectin and bone protein morphogenetic-2 ^12^. They represent non-collagen proteins found in the matrix of mineralized tissues, are considered markers of osteoblast activity, and function ^13^.

Because cigarette smoke can impair bone remodeling in a growing person, the objective of this study was to investigate the biodynamics of bone remodeling of the middle palatal suture of young rats submitted to cigarette smoke inhalation, through the analysis of osteogenic and osteoclastogenic signaling.

The hypothesis of this research is that cigarette smoke can modulate the signaling of osteoclastogenesis and osteogenesis markers in order to impair bone remodelling on middle palatal suture in rats.

## Material and methods

### Animals

In this experiment, sixty 6-week-old male Wistar rats (*Rattus norvegicus, albinus*), weighing an average of 180g, were used. This study was approved by the Ethics Committee on the Use of Animals (CEUA) of the School of Dentistry of Ribeirão Preto at University of São Paulo (FORP / USP). This study is in accordance with the ARRIVE (Animal Research: *Reporting of In Vivo Experiments*) guidelines. Throughout the experimental period, the animals were fed a standard diet and water ad libitum. A total of 60 animals were randomly assigned in groups as described on Table 1.

**Table 1:** Groups of study and periods of evaluation.

Groups	Period of Evaluation								Total
	3 days		7 days		14 days		21 days		
	H	G	H	G	H	G	H	G	
Exposed	-	5	5	5	-	5	5	5	30
Not Exposed	-	5	5	5	-	5	5	5	30
Total	10		20		10		20		60

*H= Histological Evaluations, G= Gene Expression Analysis

### Exposure to cigarette smoke

**Figure 1:**
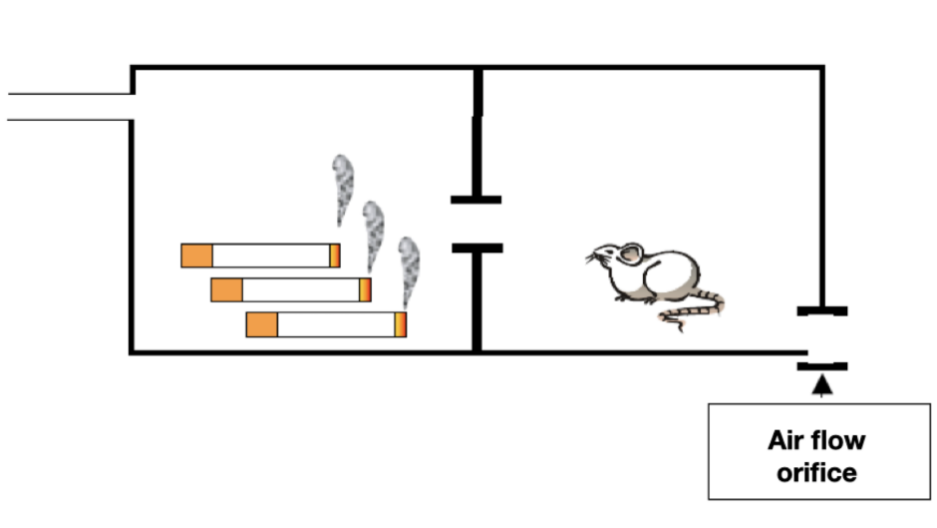
Schematic drawing representing the mechanism of exposure to smoke. It can be observed the chamber 1 where the cigarettes were positioned and the chamber 2, where the animals were kept during the exposure to cigarette smoke.

The methodology used for the exposure of animals to smoke was initially described by Le Mesurier et al. (1981) ^14^ and readapted by Nociti et al. (2002) ^15^. The chamber used for that purpose is composed of a transparent acrylic container, with dimensions of 45x25x20 cm^3^, composed of 2 chambers connected by a hole. In the first one, lit cigarettes were stored (Bonter, Ensino e Pesquisa- Equipamentos de Precisão - Ribeirão Preto-SP). In this part there is also an entrance through which air is pumped, with an inhaler (Inalar Compact NS) forming a chain that takes the smoke to the second chamber, where the animals were kept. In the second chamber, there is another orifice that provides flow to the pumped air (Figure 1).

The animals exposed to cigarette smoke were submitted to a previous adaptation period of 3 weeks ^16^ ^,^ ^17^. Then, the animals were exposed to the smoke of 5 cigarettes for two daily periods of 3 minutes each, with an interval of 12 hours between exposures. After the period of 3, 7, 14 and 21 days, the animals were euthanized and the pieces containing the middle palatal suture were collected.

Groups exposed (n= 30) or not to cigarette smoke (n= 30) were evaluated at experimental periods of 3, 7,14 and 21days to molecular analysis. To histological evaluations, five more animals were used in each groups of 7 and 21 days (n=20) as described on table 1.

### Histological procedure

The samples were fixed in 10% buffered formaldehyde for 48 hours, in individual flasks. Then, the specimens were washed in running water for 24 hours and placed in a 10% EDTA (ethylene-diamino-tetra-acetic)-based demineralizing solution, buffered in neutral pH (7.0 - 7.4), for a period of 4 weeks. After undergoing demineralization, all specimens were washed in running water for 24 hours, dehydrated in increasing concentrations of alcohols, diaphanized in xylol and included in paraffin. Semi-serial axial sections were obtained in a microtome with a thickness of 5 µm. The tissues were stained with hematoxylin and eosin.

### Microscopic evaluation

Microscopic evaluation was performed in order to describe and observe cell types during the process of osteogenesis and osteoclastogenesis in the region of the medial palatal suture after exposure to cigarette smoke in young rats. In light microscopy, histological descriptions were made. The Olympus BX61 microscopic coupled to the DP72 camera (Tokyo, Japan) was used for the capture of the images.

### Evaluation of the gene expression of osteoclastogenesis and osteogenesis mediators through real-time polymerase chain reaction (qRT-PCR)

The palatal suture tissue of each animal was collected for extraction of total ribonucleic acid (RNA) using the RNeasy Mini kit, which employs a guanidine thiocyanate extraction protocol (RNeasy® Mini, Qiagen Inc., Valencia, USA). The quality of total RNA was assessed by 1% agarose gel electrophoresis (Sigma-Aldrich Corp.) containing ethidium bromide (Sigma-Aldrich Corp.) using 1x concentrated TBE (Tris-Borate-EDTA) buffer. The purity and nucleic acid mass were analyzed using spectrophotometry in NanoDrop 1000 (Thermo Fisher Scientific Inc., Wilmington, USA) at wavelengths of 230, 260, and 280 nm. Complimentary DNA (cDNA) was synthesized from 1 μg of total RNA using random primers (High Quality cDNA Reverse Transcriptase Kits, Applied Biosystems, Foster City, CA). Next, 2-µL aliquots of total cDNA were amplified by qRT-PCR using primers for *Rank* (Rn01426423_m1) *, Rankl* (Rn00589289_m1)*, Opg (*Rn00563499_m1)*, Occ (*Rn00566386_g1), *Bsp* (Rn00561414_m1), *Opn (*Rn01449972_m1), *Onc* (Rn01470624_m1), and *Bmp2* (Rn00567818_m1). The gene for the enzyme glyceraldehyde-3-phosphate dehydrogenase (*Gapdh -* Rn00667869_m1) was used as a reference. RNAse free water was used as negative control for RT-PCR reactions. Amplification was performed under the following conditions: activation of AmpliTaq Gold Enzyme polymerase at 95° C for 2 min, followed by 40 cycles of 95° C for 1 s for DNA denaturation and 60° C for 20 s for primer annealing and polymerization. Relative quantification was performed using the Livak Method (2- ΔΔCt) and rats not exposed to cigarette smoke was considered as calibrator. The groups were compared by means of Kruskal Wallis followed by Dunn post-test (α = 0.05).

## Results

### Microscopic evaluation

Microscopic examination of the structures of the middle palatal suture region of the animals exposed or not to cigarette smoke showed histological features of bone remodeling, from 7 to 21 days. Sutural bone surface was regular, organized, slightly wavy and smooth throughout its contour, except for the presence of irrigation from the underlying medullary spaces, which penetrated the suture region as perforations in the bone surface. Covering along this bone surface, there was a thin layer of osteoid tissue with the presence of resting osteoblasts (flattened cells with nuclei parallel to the bone surface, which are not very evident). The presence of active osteoblasts (large, rounded cells with cuboidal, bulky and colored nuclei lined up on the surface of the osteoid tissue) was detected. These osteoblasts generally presented themselves in an organized manner with intense synthesis activity. However, most of these cells were inactive, covering the surface of the bone borders. The sutural space showed a uniform thickness throughout its length, occupied by fibrous sutural connective tissue, rich in fibroblasts and collagen fibers and with the presence of capillaries throughout its length. Fibroblasts were characterized by having an ovoid nucleus, clear and with an evident nucleolus. In the anterior region of the suture, the bundles of collagen fibers inserted in the bone (Sharpey fibers) were thick, with perpendicular or oblique orientations to the surface of the suture. In the middle third of the suture, bundles of fibers with varied and sagittal orientations were observed, which mixed with the sutural connective tissue. In the posterior region of the suture, these collagen fibers extended from one surface to the other of the bone, in such a way that it was possible to verify the clear connection between the edges of the suture. A large number of fibrocytes, few fibroblasts and thin collagen fibers with no defined orientation were found. In the region of the suture center, most of the fibers were inserted perpendicular to the bone and projected towards the center of the sutural space in the most diverse directions, forming a tangle of fibers. Blood vessels generally followed the orientation of the bundles of collagen fibers. The vascular lumen was circular or slightly oval and some vessels were filled with blood cells.

In the premaxilla bone, medullary cavities and Haversian channels could be seen in its lateral portion. In its medial portion, the trabeculae were parallel to the midline and separated by lines of bone apposition. The layer of mature bone tissue (lamellar or secondary bone tissue) was narrow when compared to the layer of immature bone tissue (non-lamellar or primary), which was surrounding the lateral edges of the medial palatal suture. In addition, the presence of numerous osteocytes was noted within their gaps. Havers' systems were characterized by being organized and surrounded by small medullary cavities, filled with fibrous connective tissue. Each Haversian system was characterized by numerous concentric lamellae, grouped around the narrow axial canal, containing blood vessels and a small amount of loose connective tissue. The main difference observed in the group exposed to cigarette smoke was the presence of a greater range of immature bone tissue when compared to the non exposed animals.

### Gene expression

**Figure 2:**
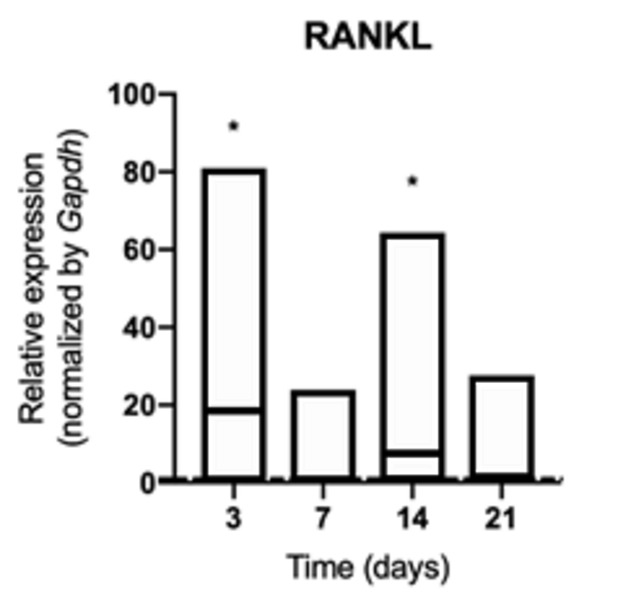
mRNA for RANKL evaluated at 3, 7, 14, and 21 days in middle palatal suture of young rats exposed to cigarette smoke. *p < 0.05 compared to the basal production of target genes in non-exposed rats (dashed line).

**Figure 3:**
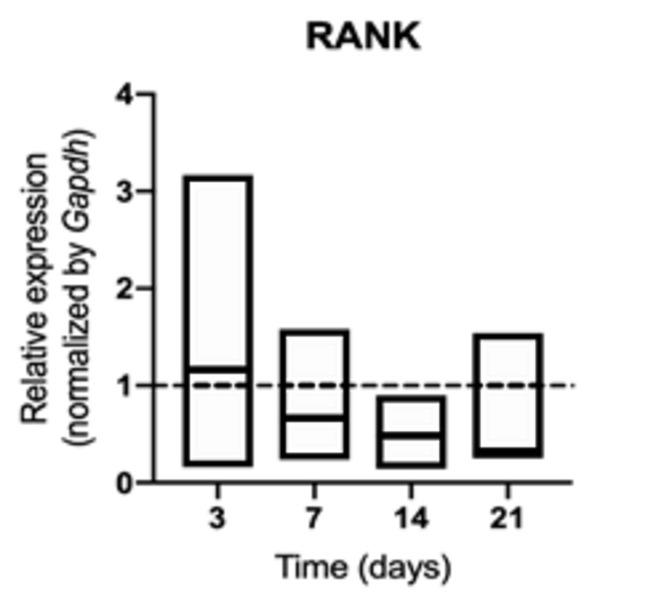
mRNA for RANK evaluated at 3, 7, 14, and 21 days in middle palatal suture of young rats exposed to cigarette smoke. *p < 0.05 compared to the basal production of target genes in non exposed rats (dashed line).

**Figure 4:**
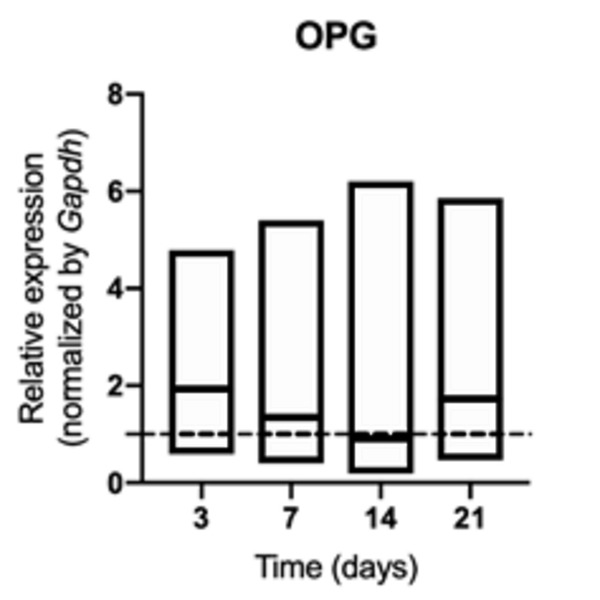
mRNA for OPG evaluated at 3, 7, 14, and 21 days in middle palatal suture of young rats exposed to cigarette smoke. *p < 0.05 compared to the basal production of target genes in non-exposed rats (dashed line).

**Figure 5:**
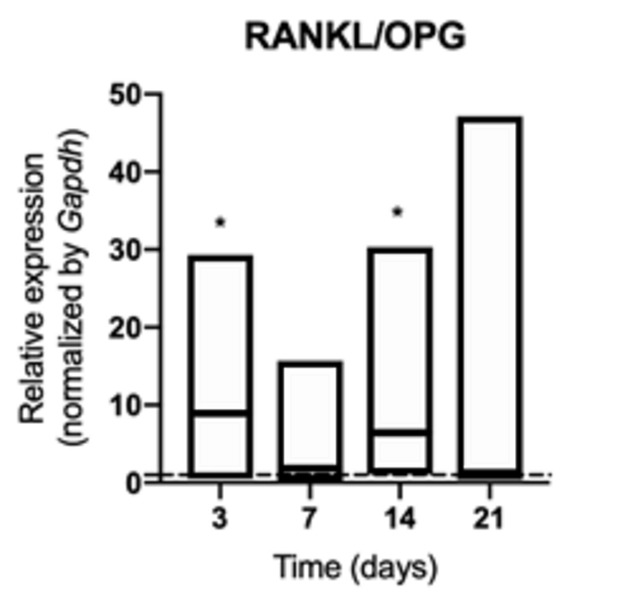
mRNA for RANKL/OPG ratio evaluated at 3, 7, 14, and 21 days in middle palatal suture of young rats exposed to cigarette smoke. *p < 0.05 compared to the basal production of target genes in non-exposed rats (dashed

Osteoclastogenic signaling was increased in animals exposed to cigarette smoke and was characterized by a higher production of RANKL at 3 and 14 days (p <0.05) (Figure 2), with no change in the synthesis of RANK (Figure 3) and OPG (p> 0.05) (Figure 4). The increased RANKL/OPG ratio showed a pro-resorptive signaling at 3 and 14 days (Figure 5).

**Figure 6:**
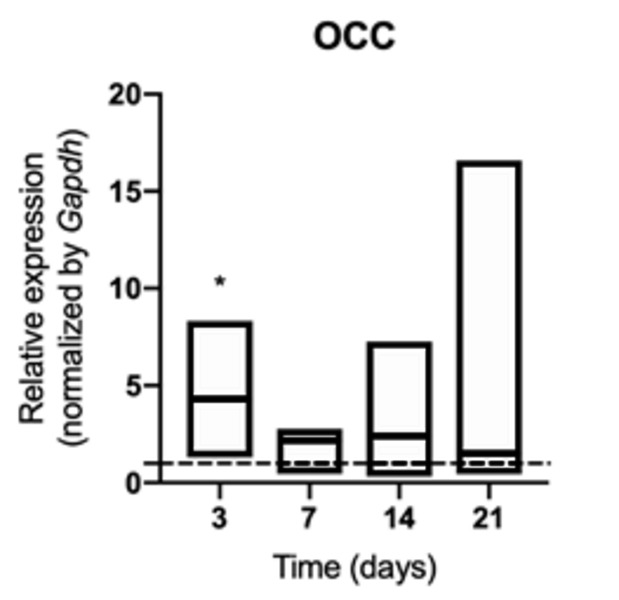
mRNA for OCC evaluated at 3, 7, 14, and 21 days in middle palatal suture of young rats exposed to cigarette smoke. *p < 0.05 compared to the basal production of target genes in non-exposed rats (dashed line).

**Figure 7:**
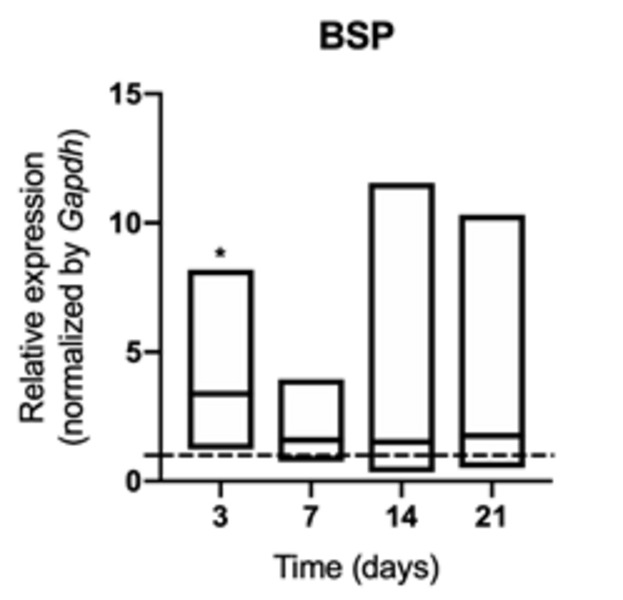
mRNA for BSP evaluated at 3, 7, 14, and 21 days in middle palatal suture of young rats exposed to cigarette smoke. *p < 0.05 compared to the basal production of target genes in non-exposed rats (dashed line).

**Figure 8:**
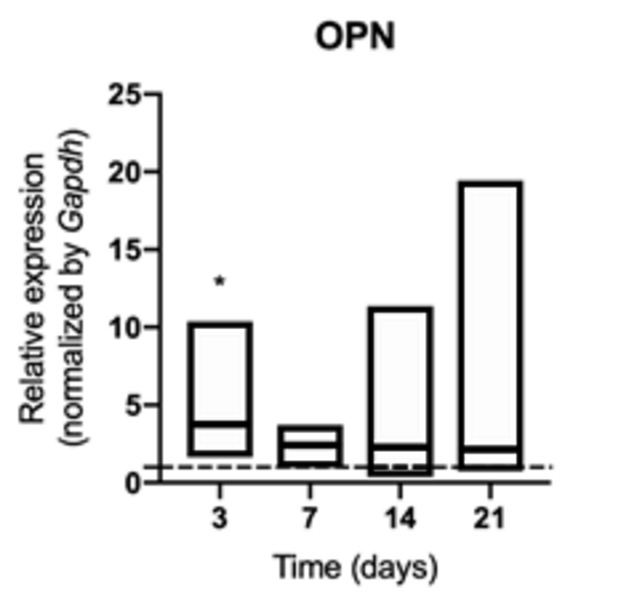
mRNA for OPN evaluated at 3, 7, 14, and 21 days in middle palatal suture of young rats exposed to cigarette smoke. *p < 0.05 compared to the basal production of target genes in non-exposed rats (dashed line).

**Figure 9:**
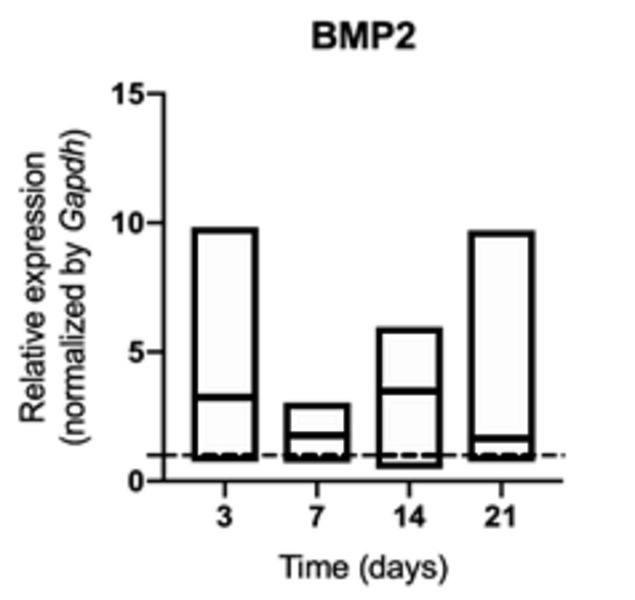
mRNA for BMP2 evaluated at 3, 7, 14, and 21 days in middle palatal suture of young rats exposed to cigarette smoke. *p < 0.05 compared to the basal production of target genes in non-exposed rats (dashed line).

**Figure 10:**
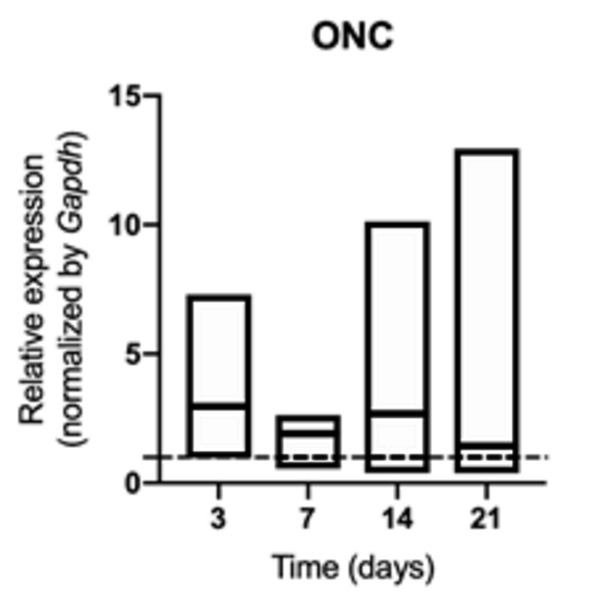
mRNA for ONC evaluated at 3, 7, 14, and 21 days in middle palatal suture of young rats exposed to cigarette smoke. *p < 0.05 compared to the basal production of target genes in non-exposed rats (dashed line).

An early increase in the synthesis of osteocalcin (Figure 6), bone sialoprotein (Figure 7) and osteopontin (Figure 8) was identified at 3 days of exposure (p <0.05) in animals exposed to cigarette smoke, although it was not sustained over time (p> 0.05). Synthesis of BMP2 (Figure 9) and ONC (Figure 10) was not changed in middle palatal suture of young rats, regardless of exposure to cigarette smoke or not (p> 0.05) (Figure 3).

## Discussion

There is a complexity of signals that can modulate the deposition of different proteins in tissues and cells. Thus, it is important to study several mediators related to bone metabolism for the identification of mediators that modulate the process of osteogenesis and osteoclastogenesis in the middle palatal suture of young animals subjected to cigarette smoke inhalation. A study with this purpose has great potential to contribute to a better understanding of the impact of this smoke in the oral cavity throughout life.

We used five cigarettes in the chamber to expose the animals to the smoke. The main goal in this research was to demonstrate what and how a chronic and passive smoker changes their metabolism during parental smoke habits. We found that smokes can modulate a signalling on gene expression in middle palatal suture in order to impair bone remodelling in long term. This research is the first and an initial study to clarify what are the genes involved in this mechanisms on middle palatal suture of young rats and probably in young children during some dentistry treatments.

Although no morphological change was evident in middle palatal suture of animals submitted or not to cigarette smoke until the period of 21 days, it is important to remember that is the first research about impact of cigarette smoke on middle palatal suture and the chronic effects on histological assessments would probably require an long term of evaluation. On the other hand, the transcriptional activity of connective tissue cells within the suture was modified. A higher pro-resorptive signaling was observed both at 3 and 14 days in animals exposed to cigarette smoke, which is in agreement with a previous study that showed increased levels of osteoclastogenesis mediators in gingival crevicular fluid of young cigarette users ^18^ ^,^ ^19^. The effects of nicotine on osteoclastic cell has been previously demonstrated by stimulation of porcine osteoclastic cells resorbing activity ^20^. In peripheral human bone marrow cell cultures, nicotine acted directly on human osteoclastic precursors, either inducing at low concentrations or inhibiting at high concentrations osteoclast differentiation ^21^. On precursors not stimulated with RANKL, nicotine increased tartrate resistant acid phosphatase activity and the expression of several osteoclast‐related genes, but did not significantly affect the resorbing activity. By contrast, on osteoclastic precursors engaged on a differentiation process, which ends up in mature osteoclasts, nicotine increased not only their differentiation, but also their resorption activity ^21^.

Nicotine has also an inhibitory effect on osteogenesis and angiogenesis, which can play a detrimental role in bone dynamics. Our findings show an early increase in the synthesis of osteocalcin, bone sialoprotein and osteopontin in animals exposed to cigarette smoke, although it was not sustained over time. It has been demonstrated that sub-toxic nicotine doses in osteoblast cell culture down-regulate type I collagen and upregulate osteonectin, alkaline phosphatase, runt-related transcription factor-2 and bone sialoprotein ^22^. These findings indicate a positive association between nicotine exposure and osteoblast phenotype and illustrate for the first time a mechanism whereby acute or chronic exposure to sub-toxic nicotine concentrations may affect bone formation through the impairment of growth factor signaling system and extracellular matrix metabolism. Although we did not observe any change on bone morphogenetic protein-2 in this study, nicotine presents a dose-dependent inhibitory effect on rabbit osteoblast cell proliferation and in the synthesis of transforming growth factor-β1, bone morphogenetic protein-2, platelet-derived growth factor, and vascular endothelial growth factor ^23^. Impaired bone formation in smokers can be directly attributed to a defective collagen synthesis ^24^.

According to the WHO, seven hundred million children are affected by indirect smoking, when considering the two billion people in the world who are victims of secondhand smoke. In Brazil, children account for 40% of these victims. All the effects of smoking are very similar at different ages, but because they have an organism that is still immature, children become the main victims of the toxicity of cigarette smoke ^25^. Because the effects of cigarette smoke on bone remodeling is widely unknown and detrimental findings have been reported, this report raises an important question regarding to long term impact on an individual bone mass, turnover and aging.

A limitation of this study is the period of evaluation that was performed just until 21 days after cigarette smoke inhalation. An extended periods will be important in future researches to understand the chronic effects of cigarette smoke inhalation on bone healing in middle palatal suture.

## Conclusion

Cigarette smoke modulates osteogenic and osteoclastogenic signaling in the middle palatal suture of young rats, although morphological changes have not been evidenced.
